# Smoking reduction in psychiatric inpatients is feasible: results from a 12-month prospective study

**DOI:** 10.1186/s12991-015-0043-5

**Published:** 2015-02-05

**Authors:** Ioannis Michopoulos, Emmanouil Rizos, Rossetos Gournellis, Andromachi Karvouni, Ioanna Kotsioumpa, Athanasios Douzenis

**Affiliations:** 2nd Department of Psychiatry, University General Hospital “Attikon”, Medical School, University of Athens, Rimini 1 Str, 12462 Athens, Greece

**Keywords:** Psychiatric inpatients, Smoking reduction, Nursing intervention

## Abstract

**Background:**

Despite the fact that smoking is a crucial morbidity factor among psychiatric patients, little progress has been made in order to reduce smoking during psychiatric hospitalization.

**Methods:**

We studied the smoking behaviour of patients admitted to a non-smoking psychiatric ward, after monitoring them for smoking habits and helping them cope in order to modify their smoking behaviour. For a period of 12 months, we conducted a prospective study of simple smoking avoidance measures in the 2nd Department of Psychiatry of Attikon University Hospital in Athens.

**Results:**

From 330 admitted patients, 170 (51.5%) were smokers; they were monitored for their smoking habits and encouraged by the nursing staff to reduce smoking. The mean number of cigarettes per day (CPD) at admission was 32.2 (sd 22.1) and upon discharge 14.1 (sd 14.8) (*t* = 11.7, *p* < 0.001). Most of the smokers, 142 (83.5%), managed to reduce their cigarette consumption per day. Diagnosis did not affect the reduction or increase in CPD. The only factor that predicted reduction in CPD was the female sex.

**Conclusions:**

Our findings indicate that seriously mentally ill psychiatric inpatients despite negative preconceptions and stereotypes respond well to simple measures aiming to reduce their smoking and modify their behaviour.

## Background

Smoking is more prevalent in psychiatric patients than in the general population. Data from the National Comorbidity Study in the USA has shown that psychiatric patients smoke twice as often as the general population without mental disorders. Furthermore, people with psychiatric disorders consume almost half (43%) of the cigarettes smoked in the USA. Smoking seems strongly connected with schizophrenia. Smokers who have schizophrenia consume more cigarettes, extract more nicotine per cigarette, prefer brands higher in nicotine, have higher blood nicotine levels and have lower success rates in attempts to stop smoking than smokers with no mental illness [1]. However, hospital settings do not often aim or are equipped to help patients to quit smoking, thus missing out an opportunity to support patients at risk because of smoking complications [2].

Therefore, we aimed to study the smoking behaviour of patients admitted to a non-smoking psychiatric ward, after monitoring them for smoking habits and helping them reduce the amount of cigarettes they smoke and cope with smoking cessation.

## Materials and methods

### Sample

We conducted a prospective naturalistic study of smoking avoidance measures in the 2nd Department of Psychiatry of Attikon University Hospital in Athens. This study was conducted in the adult inpatient psychiatric unit. Attikon Hospital serves the western suburbs of Athens which are the most densely populated area of the country.

For a period of 12 months, every consecutively admitted patient was included in the study. In total, 330 patients were admitted.

### Procedure

The patients’ smoking habits were monitored before admission, during their hospitalization and at discharge. Contrary to the current practice, it was decided that this new psychiatric unit would not tolerate smoking. Therefore, in our department, certain measures were applied in order to prevent patients from smoking: it was not allowed for anyone to smoke in the patients’ bedrooms or anywhere else inside the clinic, patients were not allowed to carry lighters or matches, smoking was only allowed in the yard and the nursing staff was always aware of who was smoking; as a nursing aim, the nursing staff tried to help patients avoid some cigarettes a day if possible. The patients were not offered any kind of medication (nicotine gums or patches) in order to reduce smoking. The number of cigarettes consumed daily was controlled by the nursing staff. The patients were informed that their smoking habits during their hospitalization were going to be recorded and used for research purposes. The study was approved by the Ethics Committee of the “Attikon” Hospital.

The nursing staff advised all tobacco users to reduce or quit smoking, assessed readiness and if the patient was willing to do it, and provided resources and assistance. The nursing staff assisted every smoker to a) remove tobacco products from his/her environment and monitor their use; b) get support from family and friends; c) review past reduction/quit attempts; d) anticipate challenges, including nicotine withdrawal, stress and mood states, particularly during the critical first few weeks; and e) identify reasons and benefits of reducing/quitting. If the patient was unwilling to reduce/quit at this time, the nursing staff helped to motivate the patient by identifying reasons for smoking cessation in a supportive manner, focusing on a) the indication why reducing/quitting was personally relevant, b) the positive and negative consequences, and c) the identification of potential benefits and potential barriers, and build patient’s confidence about reducing/quitting. Furthermore, the nursing staff followed an agreed protocol allowing drinking coffee to the patients who smoked twice a day only (i.e. in the morning and in the afternoon). This was done in order to help patients tolerate the dependence symptoms more easily and use cigarettes in an organized way. Coffee was substituted by fruit juices and chewing gums, thus breaking the habit of drinking coffee and smoking cigarettes and avoiding situations that could lead to smoking. Subsequently, the patient was encouraged and helped to explore alternative coping strategies (relaxation, exercise and creative pursuits). Additionally, nursing staff were always available to maintain a sustained contact reducing smoking using cognitive communication approaches. Before the patient’s discharge, the nursing staff discussed the patient’s progress and experiences so far and checked his/her attitude towards smoking.

### Statistical analysis

All statistical analyses were performed using the SPSS 21.0 statistical package. The independent or paired samples *t*-tests and chi-square test were used to determine differences between groups. The level of significance was set at 0.05.

## Results

From a total of 330 patients, 170 were smokers (51.5%). Regarding sex, 200 of the 330 patients (60.6%) (100 of the 170 smokers (58.8%) and 100 of the 160 non-smokers (62.5%)) were male. The mean age of all patients was 43.5, and the mean age of the smokers was 45.6 (no difference). The average length of hospitalization was 16.1 (sd 10.9) days. From the 170 smokers, 101 (59.4%) had made at least one attempt to quit smoking in the past. Their visitors were mostly smokers, too (61.7%). Regarding diagnosis, patients with schizophrenia smoke significantly more than patients suffering from mood disorders (83/177 vs. 56/138, *χ*^2^ = 4.8, *p* = 0.03). Concerning the smoking habits during hospitalization, only 12 of the 170 smokers (7%) admitted to smoking in the rooms or were seen to smoke in their rooms despite regulations.

The mean number of cigarettes per day (CPD) at admission was 32.2 (sd 22.1) and upon discharge 14.1 (sd 14.8). Most of the smokers, 142 (83.5%), managed to reduce their CPD, while only 28 (16.5%) raised or sustained their CPD. The mean difference was 18.07 CPD (sd 19.9). There was only one patient who did not smoke at all at the time of admittance, but was smoking (10 CPD) at discharge. The difference between CPD at admittance and CPD at discharge was statistically significant (*t* = 11.7, *p* < 0.001).

Regarding diagnosis, patients with schizophrenia and mood disorders did not differ in their reduction of CPD: 17.3 (sd 19.8) vs. 18.67 (sd 20.2) (*t* = 0.4, *p* = 0.65). Diagnosis did not affect the reduction or increase in CPD (*χ*^2^ = 2.5, *p* = 0.14; Table 1).

**Table 1:** **Characteristics of patients regarding smoking reduction**

	**Diagnosis**		***p***
	**Schizophrenia**	**Mood disorders**	
Smokers	83/177	56/138	*0.03*
Reduction CPD	17.30	18.67	0.65
Sex	Male	Female	
Smokers	100/170	70/170	0.75
Reduced smoking	52/100	90/100	*0.01*
Reduction CPD	20.6	14.3	*0.04*

*p* level of statistical significance, *CPD* cigarettes per day. In italics: if *p* < 0.05.

Concerning the difference regarding sex, females suffered more from mood disorders: 63 from 96 females vs. 37 from 74 males (*χ*^2^ = 4.2, *p* = 0.043). More females managed to reduce CPD: 90 from 100 females vs. 52 from 70 males (*χ*^2^ = 7.3, *p* = 0.01). The same was observed in the difference in CPD: 20.6 CPD for females vs. 14.3 CPD for males (*t* = 2.06, *p* = 0.041). When the difference in CDP was checked with regression analysis, it proved that the only factor that predicted reduction in CPD was the female sex.

## Discussion

This is the first study assessing inpatients’ smoking behaviour in a non-smoking psychiatric unit in Greece. In our study, we monitored the smoking habits of patients admitted at a non-smoking psychiatric department and the patients were actively encouraged to reduce their CPD. We found that with this simple intervention, most of the smokers (83.5%) managed to reduce their cigarette consumption per day. The mean difference was 18.07 CPD (sd 19.9), and this was statistically significant (*t* = 11.7, *p* < 0.001). Patients with schizophrenia and mood disorders did not differ in their reduction of CPD. The only factor that predicted reduction in CPD was the female sex.

Greece has one of the highest smoking rate in the world and the highest rate (more than 40%) in the European Union with the second highest consumption of cigarettes per day (21.4) according to the Eurobarometer (Special Survey on Tobacco 332 (EB 72.3)) [3]. At the time of the study, Greece had a specific legislation about smoking prevention, but this was too old (1979) and there were too many violations, even in hospitals. At that time, there was discussion on the pros and cons of enforcing a smoking ban [4]. This difficulty in enforcing a cigarette smoking ban is even more pronounced in psychiatric units that until now have not been able to implement a total cigarette ban since there is concern that a smoking ban will lead to an increase of violent incidents [5]. The opening of a new psychiatric inpatient unit offered a unique opportunity to study the effect of a total smoking ban on the ward with patients who had never been hospitalized in this unit.

Smoking is common among patients admitted in psychiatric units worldwide [6]. This finding is closely associated with the diagnosis of schizophrenia. Several studies have shown that after correcting for possible confounders, such as demographic and socioeconomic status, alcohol and antipsychotic use, or institutionalization, higher rates of smoking are still found in schizophrenia across cultures and countries [1,6]. It is unknown why there is such widespread smoking in patients with schizophrenia. Some evidence seems to support that nicotine may help alleviate negative symptomatology, reduce side effects of antipsychotic medications and possibly ameliorate cognitive deficits [7,8]. Our study’s findings are in concordance with the scientific literature: Patients with schizophrenia smoke significantly more than the patients suffering from mood disorders (83/177 vs. 56/138, *χ*^2^ = 4.8, *p* = 0.03).

Regarding sex differences’ effect on reducing smoking behaviour, literature findings are controversial. A Spanish population cohort study has reported that the average reduction in the number of cigarettes among subjects who reduced their tobacco consumption was similar in men and women (13 cigarettes/day) [9]. On the other hand, a Danish population study has shown that quitting smoking was positively associated with the male gender, older age, impaired lung function and being a moderate smoker (15–24 g/day) and negatively associated with inhaling the smoke [10]. Our study included only inpatients in a psychiatric unit. We found that female inpatients benefited more than males from our intervention. Our finding is in concordance with a Korean study in which females were less likely to be current smokers and more likely to be former smokers [11].

Though psychiatric inpatients smoke at high rates, interventions to reduce smoking and deal with nicotine addiction are scarce [2]. Nevertheless, there are studies that have underlined the need for cessation advice and appropriate follow-up care to psychiatric inpatient smokers [12,13]. The role of the nursing staff in helping psychiatric inpatients to avoid smoking is crucial [14,15]. It has been suggested that the reasons for these low rates of assessment and treatment may include the health professionals’ acceptance of smoking by psychiatric patients as a matter of individual rights and as a means of self-medication aimed at relieving symptoms or medication side effects. Staff generally anticipated more smoking-related problems than actually occurred. On the other hand, it has been observed that “There was no increase in aggression, use of seclusion, discharge against medical advice or increased use of as-needed medication following a smoking ban” [16]. It has been also shown that patients with schizophrenia under medication who quit smoking do not suffer deterioration in their mental health or quality of life [17]. It is also noteworthy that patients themselves and their relatives are interested in reducing the number of cigarettes that they smoke [14]. Our study showed that when the medical and nursing staff made consistent yet simple efforts in order to help patients, their smoking was substantially curtailed.

We have to report some limitations of the present study. Because of its naturalistic design, we did not include a control group (patients that were informed that smoking was not allowed but were not given any help or instructions by the nursing staff). This was not done because the therapeutic team did not want to give the message that some patients were receiving more attention and had been treated differently by the nursing staff during their hospitalization.

## Conclusions

Implementing a smoke-free inpatient environment and nursing support was enough to lead to a substantial modification of the inpatients’ smoking behaviour. Our findings indicate that seriously mentally ill psychiatric inpatients despite negative preconceptions and stereotypes are able to reduce their smoking easily without side effects with minimal intervention.
